# Whole blood transfusion in pediatric trauma patients with traumatic brain injury may decrease the risk of early death

**DOI:** 10.1007/s00383-025-06175-8

**Published:** 2025-08-27

**Authors:** Robert Painter, Jeffry Nahmias, Peter D. Nguyen, Yigit Guner, Laura F. Goodman, Patrick M. Chen, Jefferson Chen, Tyler Liang, Areg Grigorian

**Affiliations:** 1 Department of Surgery, Division of Trauma, Burns and Surgical Critical Care, University of California, Irvine, Orange, CA USA; 2 Division of Pediatric Surgery, Children’s Hospital Orange County, Orange, CA USA; 3 Department of Neurology, University of California, Irvine, Orange, CA USA; 4 Department of Neurological Surgery, University of California, Irvine, Orange, CA USA; 5 University of California, Irvine, 1001 Health Sciences Road, Irvine, CA 92697 USA

**Keywords:** Traumatic brain injury, Whole blood transfusion, Early mortality, Pediatric trauma

## Abstract

**Objective:**

There has been a resurgence in the use of whole blood for trauma resuscitation, however the outcomes for pediatric trauma patients with traumatic brain injury (TBI) resuscitated with whole blood are unknown. We hypothesized a lower risk of mortality and complications for pediatric trauma patients with TBI resuscitated with whole blood compared with those resuscitated exclusively with component blood therapy.

**Methods:**

The 2020–2021 TQIP database was queried for pediatric trauma patients (≤ 17 years-old) with TBI requiring blood product resuscitation. Multivariable analysis was performed to determine associated risk of overall and early (within 24 h) mortality and overall complications.

**Results:**

From 1740 transfused pediatric trauma patients with TBI, 195 (11%) received whole blood. The whole blood cohort received a similar amount of overall blood products and had similar overall rates of complications and death (all *p* > 0.05). After adjusting for age, injury severity score, and vitals on arrival, whole blood patients continued to have no difference in risk of complications and overall mortality (both *p* > 0.05). However, whole blood had decreased associated risk of early mortality (OR 0.49, CI 0.29–0.83, *p* = 0.008).

**Conclusions:**

This study found only 11% of pediatric trauma patients with TBI received whole blood, however whole blood patients had a lower associated risk of early death compared with component blood therapy. Future prospective research is needed to validate these findings.

## Introduction

Traumatic injuries are the leading cause of death for pediatric patients in the United States. Despite a trend towards a declining mortality rate in the early 2000s for pediatric trauma patients, recent data indicates an alarming reversal of this trend [1]. Similar to adults, hemorrhage and traumatic brain injury (TBI) are among the most common causes of death in pediatric trauma patients [2]. The combination of TBI with hypotension notably exacerbates the risk of mortality [3, 4], with one study suggesting an eight-fold increased risk with recurrent episodes of hypotension [5]. Furthermore, both TBI [6] and hemorrhage [7, 8] independently contribute to trauma induced coagulopathy. TBI results in the release of tissue factor as well as other platelet activating factors and procoagulants triggering a complex coagulation pathway that may result in a hypercoagulable or hypocoagulable state [6]. In contrast, hemorrhage contributes to coagulopathy by depleting clotting factors as well as causing endothelial release of procoagulants [7]. The ideal method of resuscitation in trauma patients with TBI is unknown.

In light of these challenges, there has been a surge in the use of whole blood in trauma patients in recent years. This shift is grounded in evidence suggesting that whole blood transfusion offers a mortality benefit for hypotensive trauma patients [8, 9]. Whole blood is characterized by elevated levels of nitric oxide and 2, 3-diphosphoglycerate [10]. This composition not only reduces vasoconstriction but also enhances oxygen delivery to tissues, possibly offering a survival advantage to patients with TBI that require blood product resuscitation [11]. In the context of pediatric trauma patients, the safety and efficacy of whole blood transfusion have been documented, with several studies also reporting a reduction in total transfusion requirements [10, 12–14]. However, the widespread adoption and effectiveness of whole blood transfusions in pediatric trauma patients with TBI are unknown.

This study aimed to understand whole blood transfusion effects on pediatric trauma patients with TBI. We hypothesize that whole blood transfusion in pediatric trauma patients with TBI requiring blood product resuscitation will reduce the associated risk of mortality and decrease overall requirements for blood transfusions in comparison to patients resuscitated solely with component blood therapy.

## Methods

### Data collection

This study was exempted by our institutional review board and a waiver of consent granted due to use of a deidentified national database. We queried the 2020–2021 Trauma Quality Improvement Program (TQIP) database for pediatric trauma patients (≤ 17 years-old) with TBI that underwent either component blood therapy (plasma, packed red blood cells [PRBC], platelets) or whole blood $\pm$ component blood therapy (which will be called the whole blood cohort) within four hours of arrival. The dataset only records blood transfusions occurring within the first four hours of arrival, so this was the only variable utilized to evaluate transfusion requirements. We excluded patients transferred from other facilities and all patients not requiring blood product resuscitation. We compared two groups: pediatric trauma patients with TBI receiving whole blood and those that received component blood therapy only. The primary outcome was mortality. Mortality was further categorized into deaths occurring in the emergency department, early death (occurring within 24 h of arrival), death after 48 h of arrival and overall death. Secondary outcomes included in-hospital complications and overall blood product transfusions (units of whole blood, PRBCs, platelets and/or plasma).

Demographic information included age, sex and comorbidities such as smoking. The injury profile included injury severity score (ISS), mechanism of injury, and concomitant injuries by organ system. Vitals on arrival were also collected including respiratory rate, systolic blood pressure and heart rate. Hypotension was defined as a systolic blood pressure ≤ 90 mmHg and tachycardia was defined by a heart rate $\ge$ 120 beats-per-minute. In-hospital complications included delirium, stroke, cardiac arrest, myocardial infarction, unplanned intubation, pulmonary embolism, ventilator associated pneumonia, acute kidney injury, deep vein thrombosis, compartment syndrome, unplanned return to the operating room, and infectious complications including catheter associated urinary tract infection, central line associated blood stream infection, organ space surgical site infection, deep surgical site infection, and incisional surgical site infection. Other metrics collected included hospital and intensive care unit length of stay, and ventilator days.

### Statistical analysis

Frequency statistics were performed on the variables listed previously. Mann–Whitney-U test and chi-square test were used to compare continuous and categorical variables, respectively. Categorical data were reported as percentages and continuous data were reported as medians with interquartile range.

A multivariable logistic regression analysis was performed to determine associated risk of mortality in pediatric trauma patients with TBI requiring blood product transfusion. Confounding variables were informed by literature review [8, 10] and consensus among coauthors and included age, ISS, mechanism of injury and vitals on arrival. Given that we were assessing the associated risk of mortality in our multivariable logistic regression analysis, we used the Hosmer–Lemeshow test as our model fit assessment tool [15]. The test demonstrated a good fit, as evidenced by a chi-square statistic of 9.39 and a *p*-value of 0.31. This suggests that there is no significant difference between the observed outcomes and the outcomes predicted by the model, indicating that the model is adequately calibrated and can be considered reliable for predicting mortality risk in this context. The associated risk of mortality was reported with an odds ratio (OR) and 95% confidence intervals (CI). We performed subset analyses in patients with blunt or penetrating only mechanisms. All p-values were two-sided with statistical significance of < 0.05. All analyses were performed with IBM SPSS Statistics for Windows (Version 29, IBM Corp., Armonk, NY).

## Results

### Demographics, comorbidities, and injury characteristics of pediatric trauma patients with TBI transfused with whole blood versus component blood therapy

From 1740 pediatric trauma patients with TBI, 195 (11%) were transfused whole blood and 1545 (89%) were transfused only component blood therapy. The whole blood cohort was older (median age, 15 vs 14 years, *p* = 0.003) with a higher proportion of males (72.0% vs 64.5%, *p* = 0.04), greater median ISS (34 vs 30, *p* = 0.003), and a higher rate of hypotension on arrival (46.8% vs 30.8%, *p* < 0.001) (Table 1). The mechanisms of injury were similar between groups except for whole blood patients having a higher rate of motorcycle collisions (4.6% vs 1.9%, *p* = 0.02) (Table 2). Cardiac injuries were more common in the whole blood group (7.2% vs 3.1%, *p* = 0.004) whereas small intestine injuries were less frequent (0.5% vs 3.6%, *p* = 0.02) when compared with the component blood therapy cohort (Table 3).

**Table 1:** Demographics and comorbidities of patients receiving whole blood versus component blood therapy

	Whole blood	Component blood	
Characteristic	(*n* = 195)	(*n* = 1545)	*p*-value
Age, year, median (IQR)	15 (5)	14 (9)	**0.003**
Smoking, n (%)	5 (2.9%)	20 (1.4%)	0.14
ISS, median (IQR)	34.0 (17)	30.0 (16)	**0.003**
Male, n (%)	136 (72.0%)	992 (64.5%)	0.04
Vitals on arrival, n (%)			
SBP $\le$ 90 mmHg	88 (46.8%)	457 (30.8%)	** < 0.001**
RR $\ge$ 22 bpm	74 (42.3%)	695 (48.5%)	0.12
HR $\ge$ 120	85 (44.5%)	745 (48.9%)	0.25

Bold values are statistically significant (*p* < .05)

*ADHD* Attention deficit hyperactivity disorder, *ISS* Injury severity score, *SBP* Systolic blood pressure, *RR* Respiratory rate, *bpm* breaths per minute, *HR* Heart rate

**Table 2:** Mechanism of injury for patients receiving whole blood versus component blood therapy

	Whole blood	Component blood	
Mechanism	(*n* = 195)	(*n* = 1545)	*p*-value
Blunt, n (%)			
Auto vs pedestrian	27 (13.8%)	187 (12.1%)	0.49
Bicycle	3 (1.5%)	54 (3.5%)	0.15
Fall	8 (4.1%)	100 (6.5%)	0.20
Motor vehicle collision	94 (48.2%)	721 (46.7%)	0.69
Motorcycle collision	9 (4.6%)	30 (1.9%)	**0.02**
Penetrating, n (%)			
Gunshot wound	48 (24.6%)	334 (21.6%)	0.34
Stab wound	0 (0.0%)	13 (0.8%)	0.20

Bold value is statistically significant (*p* < .05)

**Table 3:** Injuries profile for patients receiving whole blood versus component blood therapy

	Whole blood	Component blood	
Injury, n (%)	(*n* = 195)	(*n* = 1545)	*p*-value
Cardiac	14 (7.2%)	48 (3.1%)	**0.004**
Lung	114 (58.5%)	802 (51.9%)	0.08
Rib fracture	57 (29.2%)	356 (23.0%)	0.06
Esophagus	0 (0.0%)	0 (0.0%)	
Diaphragm	4 (2.1%)	21 (1.4%)	0.44
Kidney	21 (10.8%)	175 (11.3%)	0.82
Liver	42 (21.5%)	294 (19.0%)	0.40
Pancreas	3 (1.5%)	30 (1.9%)	0.70
Spleen	39 (20.0%)	280 (18.1%)	0.52
Stomach	0 (0.0%)	8 (0.5%)	0.31
Small intestine	1 (0.5%)	55 (3.6%)	0.02
Colon	8 (4.1%)	59 (3.8%)	0.85
Rectum	2 (1.0%)	7 (0.5%)	0.29
Bladder	6 (3.1%)	30 (1.9%)	0.29
Lower extremity fracture	50 (25.6%)	344 (22.3%)	0.29
Upper extremity fracture	28 (14.4%)	242 (15.7%)	0.64
Spine fracture	56 (28.7%)	442 (28.6%)	0.98

Bold value is statistically significant (*p* < .05)

### Outcomes and complications of pediatric trauma patients with TBI transfused with whole blood versus component blood therapy

The whole blood cohort had a similar rate of overall complications (28.2% vs 24.3%, *p* = 0.24) but a higher rate of unplanned returns to the operating room (6.2% vs 3.0%, *p* = 0.02). Whole blood patients had a similar overall mortality rate (10.3% vs 15.0%, *p* = 0.08) as well as a statistically similar early death rate (10.3% vs. 15.0%, *p* = 0.079) (Table 4). The whole blood cohort also received a similar number of overall blood products (11 vs. 10 units, *p = *0.46). However, the whole blood cohort had a longer hospital LOS (23 vs 15 days, *p* < 0.001). (Table 5).

**Table 4:** Complications and outcomes of patients who received whole blood transfusions versus component blood therapy

Outcomes, n (%)	Whole blood (*n* = 195)	Component blood (*n* = 1545)	*p*-value
Any complication	55 (28.2%)	376 (24.3%)	0.24
Complications, n (%)			
Delirium	7 (6.0%)	16 (2.0%)	**0.01**
Stroke	3 (1.5%)	25 (1.6%)	0.93
Cardiac arrest	24 (12.3%)	181 (11.7%)	0.81
Myocardial infarction	0 (0.0%)	1 (0.1%)	0.72
Intubation	4 (2.1%)	19 (1.2%)	0.35
Pulmonary embolism	2 (1.0%)	11 (0.7%)	0.63
Ventilator associated pneumonia	11 (5.6%)	42 (2.7%)	**0.03**
Acute kidney Injury	1 (0.5%)	27 (1.7%)	0.20
Deep vein thrombosis	5 (2.6%)	40 (2.6%)	0.98
Extremity compartment syndrome	0 (0.0%)	7 (0.5%)	0.35
Catheter associated urinary tract infection	1 (0.5%)	9 (0.6%)	0.90
Central line associated blood stream infection	1 (0.5%)	1 (0.1%)	0.08
Sepsis	0 (0.0%)	10 (0.6%)	0.26
Incisional surgical site infection	0 (0.0%)	12 (0.8%)	0.22
Deep surgical site infection	1 (0.5%)	5 (0.3%)	0.67
Organ space surgical site infection	2 (1.0%)	2 (0.1%)	**0.01**
Return to operating room	12 (6.2%)	46 (3.0%)	**0.02**
Mortality rates, n (%)			
Emergency department	15 (7.7%)	143 (9.3%)	0.47
Within 24 h	20 (10.3%)	231 (15.0%)	0.08
After 48 h	72 (36.9%)	464 (30.0%)	0.05
Index hospitalization	93 (47.7%)	725 (46.9%)	0.84

Bold values are statistically significant (*p* < .05)

**Table 5:** Outcomes for patients who received whole blood transfusions versus component blood therapy

Outcomes, median (IQR)	Whole blood (*n* = 195)	Component blood (*n* = 1545)	*p*-value
Hospital days	23 (10, 36)	15.0 (3, 26)	** < 0.001**
ICU length of stay	7 (0, 15)	5 (0, 10)	0.14
Ventilator days	4 (0, 8)	3 (0, 6)	0.55
Total blood products in the first 4 h	11 (2, 10)	10 (2, 10)	0.46
PRBC within 4 h	3 (1, 4)	2 (0, 3)	**0.01**
FFP within 4 h	3 (1, 5)	2 (1, 4)	0.06
Platelets within 4 h	1 (1, 1)	1 (1, 2)	**0.005**

Bold values are statistically significant (*p* < .05)

*ICU* Intensive care unit, *PRBC* Packed red blood cells, *FFP* Fresh frozen plasma

After adjusting for age, ISS, hypotension and tachycardia on arrival, whole blood patients had a similar overall associated risk of mortality (OR 0.73, CI 0.51–1.07, *p* = 0.11) as well as in-hospital complications (OR 1.04, CI 0.70–1.55, *p* = 0.85), compared with component blood therapy patients. Whole blood patients had a decreased associated risk of early death (OR 0.49, CI 0.29–0.83, *p* = 0.008) (Table 6). Subgroup analysis revealed that pediatric trauma patients with TBI who were transfused with whole blood showed no significant difference in overall associated risk of mortality when mechanisms were classified as blunt (OR 0.78, CI 0.55–1.08, *p* = 0.13) or penetrating (OR 0.79, CI 0.38–1.63, *p* = 0.52). However, whole blood patients had a decreased associated risk of early death for both blunt (OR 0.50, CI 0.29–0.84, *p* = 0.01) and penetrating injuries (OR 0.36, CI 0.14–0.97, *p* = 0.04) (Table 7).

**Table 6:** Multivariable logistic regression determining risk of various outcomes for pediatric trauma patients with TBI receiving whole blood transfusions versus component blood therapy

Risk factors	OR	CI	*p*-value
Mortality			
Emergency department	0.66	0.36–1.20	0.17
Within 24 h	0.49	0.29–0.83	**0.008**
After 48 h	1.20	0.87–1.66	0.27
Overall	0.73	0.51–1.07	0.11
Any complication	1.04	0.70–1.55	0.85

Bold value is statistically significant (*p* < .05)

^*^Controlled for age, injury severity score, hypotension and tachycardia on arrival

**Table 7:** Multivariable logistic regression determining risk of mortality outcomes for subgroups of pediatric trauma patients with TBI receiving whole blood transfusions versus component blood therapy

Risk factors	OR	CI	*p*-value
Blunt mechanism mortality			
Emergency department	0.67	0.37–1.23	0.19
Within 24 h	0.50	0.29–0.84	**0.01**
After 48 h	1.22	0.88–1.69	0.24
Overall	0.78	0.55–1.08	0.13
Penetrating mechanism mortality			
Emergency department	0.58	0.21–1.62	0.30
Within 24 h	0.36	0.14–0.97	**0.04**
After 48 h	1.77	0.92–3.42	0.09
Overall	0.79	0.38–1.63	0.52

Bold values are statistically significant (*p* < .05)

^*^Controlled for age, injury severity score, hypotension and tachycardia on arrival

## Discussion

The transfusion of whole blood has gained increasing traction [16]. This national database study reveals that whole blood transfusion is used in 11% of pediatric trauma patients with TBI receiving blood product resuscitation. Furthermore, pediatric trauma patients with TBI receiving whole blood had a lower associated risk of early death (within 24 h) when compared with patients receiving only component blood therapy. This remained true for separate subgroup analyses of both blunt and penetrating trauma patients. This finding highlights the potential role of whole blood transfusion in pediatric trauma care and emphasizes the need for further research, especially among specific groups such as those with TBI to improve treatment strategies.

TBI independently contributes to trauma induced coagulopathy and mortality in pediatric trauma patients [4, 6]. Children face an elevated risk of intracranial hypertension following severe TBI, making them more vulnerable to secondary brain injury [17]. The inherent properties of whole blood transfusion, notably it’s increased hematocrit may offer protective qualities against cerebral edema while simultaneously enhancing oxygen delivery [10]. This dual benefit may mitigate secondary brain insults for pediatric trauma patients with TBI. Additionally, TBI patients are particularly vulnerable to hypotension, often exacerbated by impaired autonomic regulation, which poses a significant challenge in ensuring sufficient cerebral perfusion [11]. The comprehensive nature of whole blood, containing all blood components in their natural ratios, could more effectively restore the blood’s oxygen-carrying capacity and coagulation factors. This is especially vital in the acute phase following trauma, where resuscitation can prevent the cascade of hypoperfusion and secondary brain injury. Moreover, whole blood transfusions may minimize the exposure to different donors, potentially reducing the risk of alloimmunization, transfusion-related complications and transfusion-transmitted infections [18]. Additionally, the enhanced hemostatic properties of fresh whole blood might be particularly advantageous in managing TBI-related bleeding, offering a more immediate and effective correction of coagulopathy.

Pediatric trauma patients with TBI face the highest risk of mortality within the first 24 h, a period during which TBI is the most common cause of death [19]. In this context, the early correction of coagulopathy and hypotension is important for improving outcomes in pediatric trauma patients with TBI. Whole blood transfusions can significantly enhance the efficiency of emergency resuscitation by reducing the logistic complexity associated with administering multiple blood components and more effective correction of trauma induced coagulopathy. This study found that pediatric trauma patients with TBI who underwent whole blood resuscitation exhibited a lower associated risk of early death compared with those receiving solely component blood therapy. While this retrospective analysis cannot conclusively attribute the survival advantage to whole blood, the association warrants further investigation. Furthermore, Hanna et al.'s work, categorizing adult trauma cases by the mechanism of injury, noted a decrease in early mortality for both blunt and penetrating injuries [8]. Our study extends these observations to pediatric patients with TBI and supports further adoption of pediatric whole blood resuscitation in the setting of TBI.

Pediatric trauma patients who experience TBI events have a higher risk of developing psychological, social, and cognitive impairments into adulthood [20]. Additionally, pediatric trauma patients may be more vulnerable to negative long-term outcomes when compared with adults with similar injuries [21]. This highlights the need for early intervention and correction of whole blood has shown faster balanced resuscitation than component blood therapy in pediatric trauma patients which may attenuate secondary brain injury by restoring intravascular volume [22]. Trauma patients are particularly sensitive to timely interventions with studies showing mortality benefit for early whole blood transfusion [23]. Furthermore, despite a similar hemodynamic response, one animal model showed better short term neurologic recovery with the use of whole blood compared with component blood therapy [24]. However, it is clear that long-term studies for neurologic recovery after whole blood transfusion for TBI patients are warranted. We propose that combination of higher hematocrit, better oxygen delivery, and faster resuscitation with whole blood transfusion will all contribute to an attenuated secondary brain injury.

Owing to the nature of the retrospective database design, this study is limited by selection bias as well as missing data and/or misclassification errors. Although we controlled for confounding variables that are available in the dataset, it is also possible that unaccounted differences between the cohorts could have affected our findings. In addition, the TQIP database lacks pertinent variables including ongoing physiologic data, reason for death, and details of whole blood transfused. In addition, whole blood augmented by component blood therapy may blunt some of the benefits of exclusive whole blood administration [25]. There also is significant variation in whole blood usage between trauma centers including storage time and volumes infused [26, 27]. Also, due to previously stated physiological differences, there may be variability even within the pediatric population. While our cohort is predominantly adolescents, very young children may respond differently to whole blood, which merits future research.

## Conclusions

This national analysis spanning two years of data demonstrated that whole blood use in pediatric trauma patients with TBI was associated with a reduced associated risk of early death compared with the exclusive use of component blood therapy. This persisted on both subgroup analyses of blunt and penetrating trauma patients. While the benefits of whole blood may still be incompletely understood in children, a growing body of evidence suggests that the use of whole blood in select patients may improve outcomes. With the growing adoption of whole blood in clinical practice, future prospective randomized trials are needed to establish the efficacy of a whole blood-first strategy in resuscitation protocols for pediatric trauma patients with TBI.

## Data Availability

No datasets were generated or analyzed during the current study.
